# A Biomimetic Approach toward Enhancing Angiogenesis: Recombinantly Expressed Domain V of Human Perlecan Is a Bioactive Molecule That Promotes Angiogenesis and Vascularization of Implanted Biomaterials

**DOI:** 10.1002/advs.202000900

**Published:** 2020-06-14

**Authors:** Xiaoting Lin, Fengying Tang, Shouyuan Jiang, Heba Khamis, Andre Bongers, John M. Whitelock, Megan S. Lord, Jelena Rnjak‐Kovacina

**Affiliations:** ^1^ Graduate School of Biomedical Engineerin University of New South Wales Sydney NSW 2052 Australia; ^2^ Comparative Pathology Program Department of Comparative Medicine University of Washington School of Medicine Seattle WA 98195 USA; ^3^ Biological Resources Imaging Laboratory University of New South Wales Sydney NSW 2052 Australia

**Keywords:** angiogenesis, biomaterials, biomimetic growth factor, ischemia, perlecan, silk, vascularization

## Abstract

Angiogenic therapy involving delivery of pro‐angiogenic growth factors to stimulate new blood vessel formation in ischemic disease is promising but has seen limited clinical success due to issues associated with the need to deliver supra‐physiological growth factor concentrations. Bio‐inspired growth factor delivery utilizing the native growth factor signaling roles of the extracellular matrix proteoglycans has the potential to overcome many of the drawbacks of angiogenic therapy. In this study, the potential of the recombinantly expressed domain V (rDV) of human perlecan is investigated as a means of promoting growth factor signaling toward enhanced angiogenesis and vascularization of implanted biomaterials. rDV is found to promote angiogenesis in established in vitro and in vivo angiogenesis assays by potentiating endogenous growth factor signaling via its glycosaminoglycan chains. Further, rDV is found to potentiate fibroblast growth factor 2 (FGF2) signaling at low concentrations that in the absence of rDV are not biologically active. Finally, rDV immobilized on 3D porous silk fibroin biomaterials promotes enhanced vascular ingrowth and integration of the implanted scaffolds with the surrounding tissue. Together, these studies demonstrate the important role of this biologically active perlecan fragment and its potential in the treatment of ischemia in both native and bioengineered tissues.

## Graphical abstract

A biomimetic growth factor delivery approach utilizing the native signaling roles of extracellular matrix is described. C‐terminal region of perlecan (rDV) is shown to promote angiogenesis by signaling endogenous and exogenous growth factors at low concentrations in its soluble form and when immobilized on a biomaterial, making it a promising approach toward angiogenic therapy for the treatment of ischemic disease.

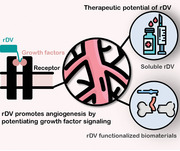

## 1 Introduction

Blood vessels allow exchange of oxygen, carbon dioxide, nutrients, and waste at the tissue level and provide conduits for transport of hormones and components of the immune system throughout the body. Inadequate or aberrant vascular supply is a hallmark of many disorders characterized by tissue ischemia, including myocardial infarction, stroke, and chronic wound healing.^[^ ^1^ ^]^ Lack of sufficient and timely vascularization is also a key obstacle in translating advances in tissue engineering and regenerative medicine to clinical applications. Thick, critically sized (greater than passive diffusion limit of oxygen, ≈500 µm thick) bioengineered tissues do not vascularize in a timely manner to allow tissue survival and integration once implanted, often resulting in cell necrosis and tissue rejection. Vascularization of implanted constructs is also important in supporting construct innervation and thus reestablishing tissue function.^[^ ^2a,b^ ^]^

The vascular network in the early embryo is established via vasculogenesis and angiogenesis. De novo blood vessel formation in the early embryo occurs via vasculogenesis, involving assembly of mesoderm‐derived endothelial precursors into the vascular tree, while in the adult, new vessel formation can occur via splitting of existing vessels (intussusception), stimulation of vessel expansion by circulating precursor cells, or via angiogenesis^[^ ^1^ ^]^. Angiogenesis, or the formation of blood vessels by sprouting from preexisting vasculature, is an essential physiological process crucial in growth and development, reproduction, and wound healing. Angiogenesis is thought to account for most new vessel formation in adult tissue, including vessel ingrowth into implanted biomaterials or bioengineered tissues (in the absence of a primitive vascular network formed in vitro).^[^ ^3^ ^]^

Angiogenesis involves stimulation of endothelial cells by pro‐angiogenic signals (such as vascular endothelial growth factor, VEGF) to form new vessels by a highly coordinated process involving degradation of the basement membrane, endothelial cell migration, proliferation, fusion, and vessel maturation and remodeling.^[^ ^1^ ^]^ As a result of the well‐established role of pro‐angiogenic growth factors like VEGF in this process, one of the earliest strategies toward vascularization of ischemic tissues or tissue engineered constructs involved delivery of pro‐angiogenic growth factors.^[^ ^4a,b^ ^]^ However, efforts to therapeutically stimulate angiogenesis using growth factors were met with limited clinical success. Difficulties associated with temporal control of growth factor delivery, as well as the short half‐life requiring delivery of supra‐physiological concentrations, and costs associated with their manufacture, limit the use of this approach as a sole means of promoting tissue vascularization.^[^ ^4^, ^5^ ^]^

Bioinspired approaches that mimic the natural storage and signaling of growth factors can potentially circumvent some of the issues associated with growth factor delivery.^[^ ^6^ ^]^ A number of interesting approaches toward this end have been explored, including delivery of multimeric complexes comprising insulin‐like growth factors (IGF), IGF‐binding proteins, epidermal growth factor (EGF), and vitronectin domains that decreased wound surface area in a pilot study for the treatment of human chronic venous leg ulcers.^[^ ^7^ ^]^ Similarly, a protein engineering approach of VEGF‐A and platelet‐derived growth factor (PDGF)‐BB has been performed to enhance their binding to extracellular matrix (ECM) molecules such as fibrinogen to enable longer residence time in the wound site and resulted in improved granulation tissue formation and vascularization.^[^ ^8^ ^]^ To overcome the challenges of limited diffusion of topical administration and rapid degradation, gene delivery approaches have also been explored to induce VEGF‐A expression using plasmid DNA delivered via a collagen/chitosan scaffold and found to promote vascularization.^[^ ^9a,b^ ^]^ Each of these bioinspired approaches is promising, but they do not mimic the biological presentation, protection, and signaling of growth factors.^[^ ^6^ ^]^

Many growth factors involved in angiogenesis naturally bind to and are potentiated by heparan sulfate (HS) glycosaminoglycan (GAG) chains that decorate proteoglycans present in the ECM and on the cell surface.^[^ ^10^ ^]^ These highly sulfated linear polysaccharides bind a range of growth factors and cytokines, and play a role in their function, by protecting them against proteolysis, allowing formation of signaling gradients, and acting as co‐receptors for growth factor signaling.^[^ ^11a,b^ ^]^ With the exception of hyaluronan, GAGs are attached to a protein core of proteoglycans, but due to challenges in their isolation and expression, proteoglycans have seen limited therapeutic applications.^[^ ^6^ ^]^

**Figure 1:**
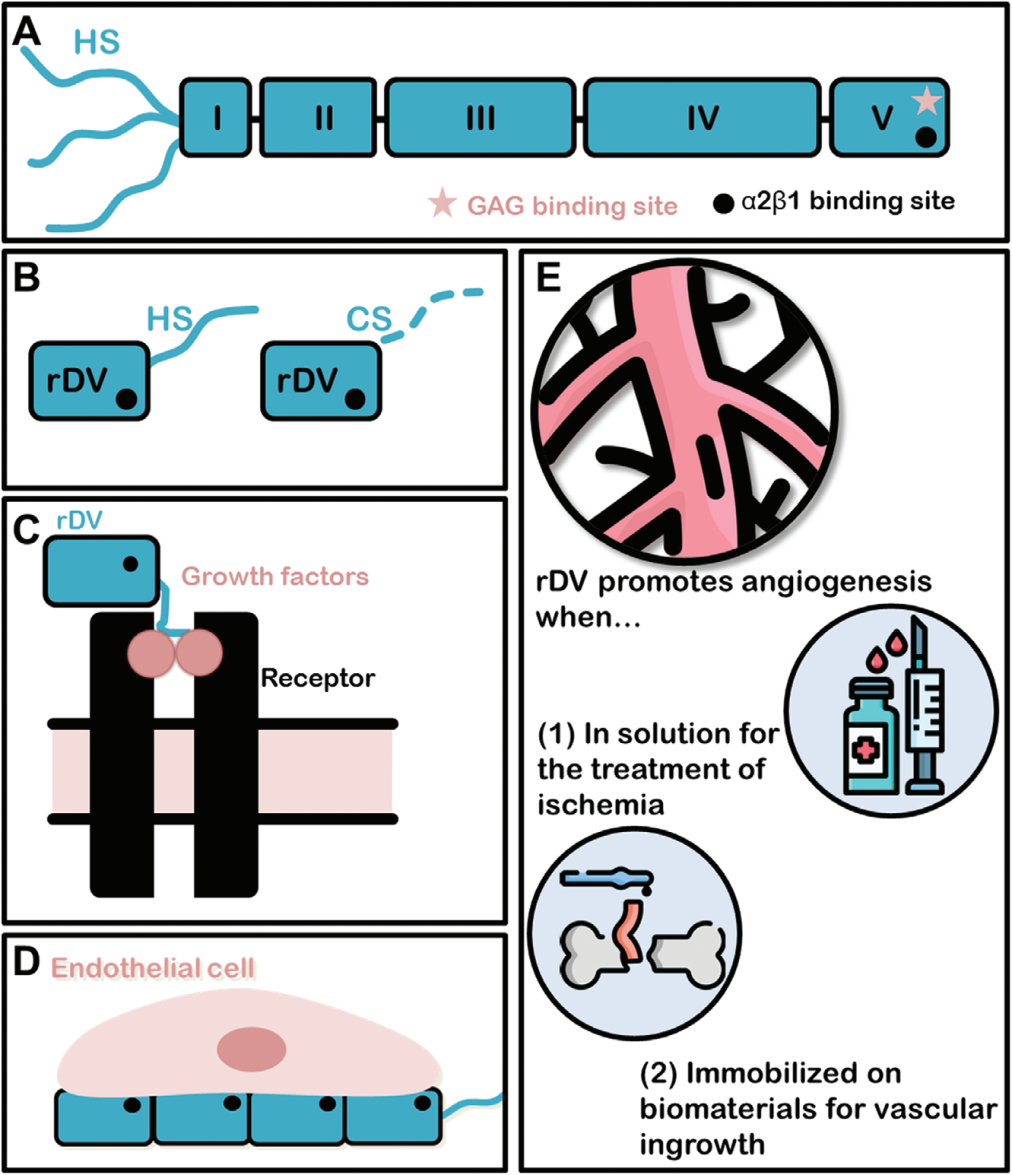
Schematic representation of the structure and function of perlecan and recombinantly expressed C‐terminal domain V region (rDV). A) Schematic representation of endothelial cell perlecan showing five protein domains and three heparan sulfate (HS) glycosaminoglycan (GAG) chains attached at domain I (N‐terminus). Another potential GAG binding site (*) is shown on Domain V (C‐terminus), which is not decorated in human endothelial cell perlecan. An *α*2*β*1 integrin binding site (●) is present on domain V and supports endothelial cell binding.^[^ ^21^ ^]^ B) Schematic representation of recombinantly expressed rDV. rDV is expressed by HEK‐293 cells as a mixed protein population consisting of an 80 kDa protein decorated with a single HS or CS GAG chain, and contains the integrin binding site (●).^[^ ^19^ ^]^ C,D) Schematic representation of rDV's roles in angiogenesis and vascularization including binding and potentiating signaling of FGF2 by forming a ternary complex with FGF2 and its cognate receptors (C), current work) and supporting endothelial cell interactions, including cell adhesion and migration (D), current work and ref. [19]. E) Potential therapeutic uses of rDV, including as a soluble factor delivered to ischemic tissue to support angiogenic therapy, or immobilized on biomaterials to support vascularization and integration of implanted biomaterials and bioengineered tissues.

Perlecan (**Figure** **1**A) is the major extracellular HS proteoglycan expressed in the vascular basement membrane. Perlecan is known to support angiogenesis though its HS chains, by binding and signaling key vascular growth factors (VEGF and fibroblast growth factor (FGF) families), while the protein core supports *α*2*β*1 integrin‐mediated endothelial cell binding.^[^ ^6^, ^12^ ^]^ Mice lacking HS chains attached to the N‐terminal domain I of perlecan have delayed wound healing and impaired angiogenesis,^[^ ^13^ ^]^ while chronic venous ulcers have reduced perlecan expression compared to normal skin.^[^ ^14^ ^]^ Perlecan has previously been coated onto vascular grafts and in vivo studies indicated superior performance compared to the pristine expanded polytetrafluoretheylene graft in terms of enhanced endothelialization and a reduction in both platelet adhesion and activation.^[^ ^15^ ^]^ While these studies indicate the biological activity of perlecan is promising for vascularization applications, the yield of perlecan from natural sources is too low for therapeutic use. Its 460 kDa protein core and extensive post‐translational modification with glycosaminoglycans makes its recombinant expression prohibitive. However, recent advances in recombinant expression and metabolic engineering approaches have enabled the production of proteoglycans or their bioactive domains^[^ ^16^ ^]^ with functional GAG chains, thus allowing better recapitulation of the native growth factor signaling events involved in biological processes, including angiogenesis.

The C‐terminal domain V region of perlecan has both anti‐angiogenic^[^ ^17a,b^ ^]^ and pro‐angiogenic ^[^ ^18a,b^ ^]^ activities depending on its source and presentation. A human domain V sequence encompassing Glu3687 to Ser4391 produced a GAG‐free protein form of domain V, termed endorepellin. In its soluble form endorepellin has been demonstrated to be anti‐angiogenic in endothelial cell and mouse tumor models, but conversely was pro‐angiogenic in the brain following ischemic injury.^[^ ^18b^ ^]^ We have expressed a slightly longer human perlecan C‐terminal sequence (Leu3626 to Ser4391) that results in a recombinant proteoglycan form of domain V (rDV)^[^ ^12^, ^18^, ^19^ ^]^ and is investigated in the current study. rDV (Figure 1B) is expressed as a proteoglycan consisting of an 80 kDa protein core containing one GAG attachment site that is decorated with either HS or chondroitin sulfate (CS).^[^ ^19^ ^]^ rDV was shown to support endothelial cell adhesion and proliferation on both tissue culture plastic or when immobilized on silk biomaterials.^[^ ^19^, ^20^ ^]^ In this work, we demonstrate the potential of rDV in promoting angiogenesis in its soluble form and vascularization of implanted biomaterials when immobilized on the biomaterial surface, with potential applications in the treatment of ischemic disease, and integration and survival of bioengineered tissues (Figure 1C–E). The ability to produce rDV as a recombinant molecule provides higher yields,^[^ ^19^ ^]^ and control and scale up of the manufacturing process that cannot be achieved when isolating full‐length perlecan from native cells or tissue, thus providing a more accessible regulatory and commercial pathway for its use in therapeutic applications.

## 2 Results

### 2.1 rDV Promotes Endothelial Cell Migration and Angiogenic Sprouting In Vitro

**Figure 2:**
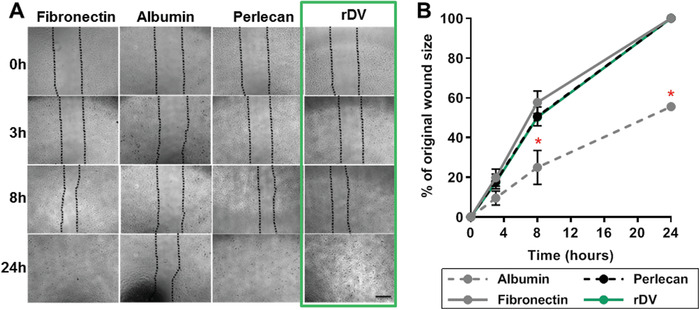
rDV promotes endothelial cell migration. HUVEC migration following a scratch assay on tissue culture plastic coated with albumin (negative control), fibronectin and full length perlecan (positive controls), and rDV over a 24 h period. A) Representative phase contrast images of HUVEC migration immediately following wound formation in the scratch assay (0 h) and at 3, 8, and 24 h following wounding. Dotted lines indicate the cell migration front. Scale bar is 100 µm. B) Quantitative analysis of HUVEC migration presented as percent of the original wound area quantified from phase contrast images. Data are mean ± SD (*n* = 4). * indicates statistically significant differences (*p* < 0.05) compared to the positive control fibronectin.

The effect of rDV on endothelial cell migration and sprouting, key processes in the formation of new blood vessels via angiogenesis, was studied in vitro. Endothelial cell monolayers established on tissue culture plastic were scratched and cell migration into the scratched “wound” area was observed compared to fibronectin and full‐length human perlecan (positive controls), and bovine albumin (negative control). Endothelial cells started migrating into the “wound” area by 3 h post‐injury and between 8 and 24 h post‐injury cells had covered the entire scratch area on fibronectin, perlecan, and rDV, but not on albumin (**Figure** **2**A). When the “wound” size was quantified (Figure 2B), on rDV it showed 17.3 ± 2.1% coverage at 3 h, 50.2 ± 1.0% coverage at 8 h, and complete coverage at 24 h, similar to that on positive controls (*p* > 0.05). While there is a risk of coated proteins in migration assays being scratched off and replaced with proteins found in cell culture media during wound creation, this effect would be expected on the albumin control as well, which was not observed here, supporting the integrity of the assay performed.

**Figure 3:**
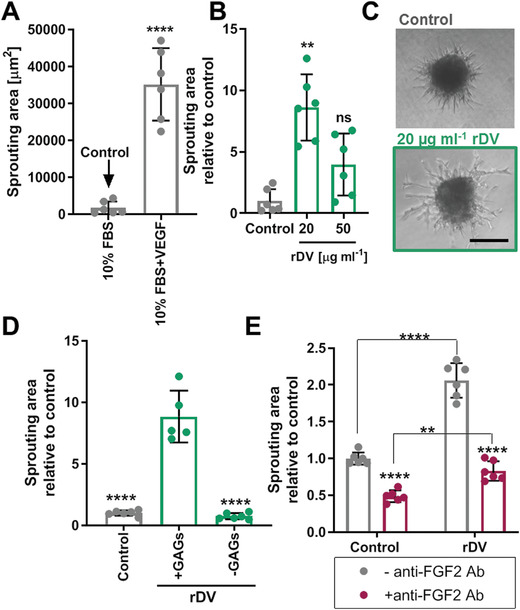
rDV promotes endothelial cell angiogenic sprouting. HUVEC spheroids were embedded in a fibrin gel and cell sprouting from the spheroid body was analyzed from phase contrast micrographs after 24 h. A) Establishment of an appropriate negative control, demonstrating that little HUVEC sprouting occurred in the presence of 10% FBS, compared to 10% FBS with 5 ng mL^−1^ VEGF165, making 10% FBS an appropriate negative control (Control). All other experimental conditions contained 10% FBS in addition to the test substance. B) HUVEC sprouting in the presence of 20 and 50 µg mL^−1^ rDV. Cell sprouting is expressed as fold change relative to Control and **p < 0.01 is relative to Control. C) Representative images of HUVEC spheroid under Control conditions or in the presence of 20 µg mL^−1^ rDV. Scale bar is 200 µm. D) HUVEC sprouting in the presence of 20 µg mL^−1^ rDV (+GAGs) or with 20 µg mL^−1^ rDV with GAG chains removed by enzymatic digestion (‐GAGs). Cell sprouting is expressed as fold change relative to Control and ****p < 0.0001 is relative to rDV+GAGs condition. E) HUVEC sprouting under Control or 20 µg mL^−1^ rDV in the absence (‐anti‐FGF2 Ab) or presence (+anti‐FGF2 Ab) of the anti‐FGF2 antibody, demonstrating that cell sprouting involves FGF2 signaling. Cell sprouting is expressed as fold change relative to Control and ****p < 0.0001 is relative to the corresponding anti‐FGF2 Ab condition unless indicated otherwise. Data are mean ± SD (*n* = 5–6).

To study the effect of rDV on endothelial cell sprouting, endothelial cells were formed into spheroids and embedded in a fibrin matrix in the presence of 10% FBS (Control). No sprouting was observed in the absence of FBS (data not shown). VEGF165 or rDV were added to embedded spheroids and endothelial cell sprouting from the spheroid body was observed and quantified after 24h. VEGF165 promoted a 20‐fold increase in the spheroid sprouting area relative to control (*p* < 0.0001) and served as a positive control for the assay (**Figure** **3**A). rDV increased endothelial sprouting by 8.6‐fold at 20 µg ml^−1^ (*p* < 0.01) and fourfold at 50 µg mL^−1^ (*p* > 0.05) compared to the control (Figure 3B). Representative images of endothelial cell spheroids under control and 20 µg mL^−1^ rDV conditions are shown in Figure 3C and illustrate the larger overall sprouting area and longer sprouts in the presence of rDV. It was hypothesized that rDV promotes endothelial cell sprouting by potentiating growth factor signaling via its GAG chains. To test this hypothesis, GAG chains were enzymatically digested from the rDV protein core before rDV was added to embedded endothelial cell spheroids for 24 h. Removal of the GAG chains significantly reduced the ability of rDV to promote endothelial cell sprouting (*p* < 0.0001) (Figure 3D), demonstrating that rDV promotes sprouting via its GAG chains. To establish that rDV promotes endothelial cell sprouting by potentiating growth factor signaling, an anti‐FGF2 antibody was added to the spheroid cultures in the absence (Control) or presence (rDV) of rDV. The antibody significantly reduced the spheroid sprouting area in both conditions (*p* < 0.0001), with a 2.5‐fold decrease in the spouting area in the rDV condition (Figure 3E). This indicates that endothelial cell sprouting involves FGF2 signaling.

### 2.2 rDV Promotes Angiogenesis In Vivo by Potentiating Growth Factor Signaling via Its GAG Chains

**Figure 4:**
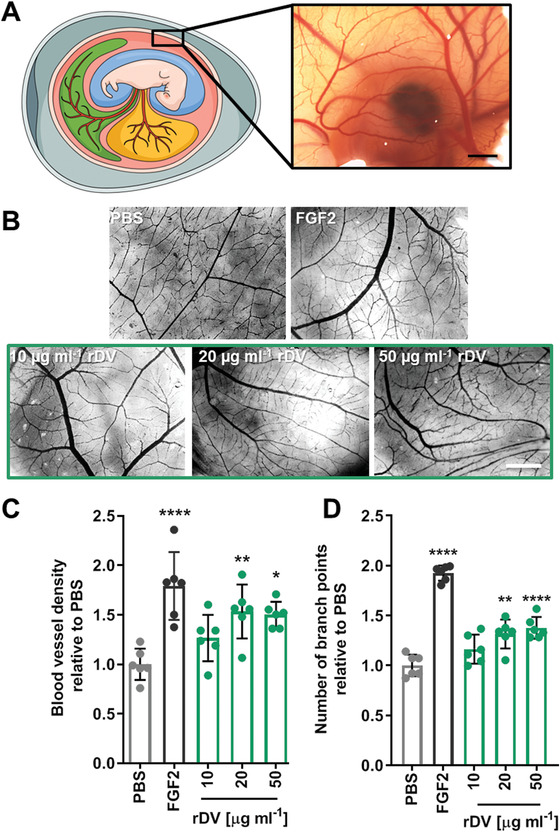
rDV promotes angiogenesis in vivo. rDV was added to the CAM at embryonic day 8 (E8) and the effect on the vessel density and the number of branch points was studied at E12. A) Schematic representation (left) of the CAM assay showing a fertilized chicken egg surrounded by a highly vascularized CAM and a dissecting microscope micrograph (right) of the CAM at E12. B) Representative dissecting microscope images of the blood vessels in the CAM at E12 following exposure to PBS (negative control), FGF2 (50 ng mL^−1^, positive control), and rDV (10, 20, or 50 µg mL^−1^). Scale bar is 1 mm. C) Blood vessel density and D) number of branch points in CAM membranes exposed to PBS, FGF2 (50 ng mL^−1^) or rDV (10, 20, or 50 µg mL^−1^). Data are expressed as fold change relative to PBS. Data are mean ± SD (*n* = 6). *p < 0.05, **p < 0.01, and ****p < 0.0001 relative to PBS.

The effect of rDV on angiogenesis in vivo was studied in a CAM angiogenesis assay (**Figure** **4**A). rDV was added to the CAM surface and the effect on blood vessel density and the number of blood vessel branch points was assessed 4 days later at embryonic day 12 (Figure 4B). rDV showed a concentration‐dependent effect on angiogenesis, with 10 µg mL^−1^ rDV showing no effect on the blood vessel density or number of branch points relative to the negative control PBS (*p* > 0.05), but both 20 and 50 µg mL^−1^ significantly increased both measures of angiogenesis compared to PBS (Figure 4C,D). rDV at 20 and 50 µg mL^−1^ increased blood vessel density by twofold and 2.5‐fold, respectively, and increased the number of branch points by 1.3‐fold and 1.4‐fold, respectively, compared to PBS. Both angiogenesis measures for rDV (20 and 50 µg mL^−1^) were not significantly different to those of the positive control FGF2 (*p* > 0.05).

**Figure 5:**
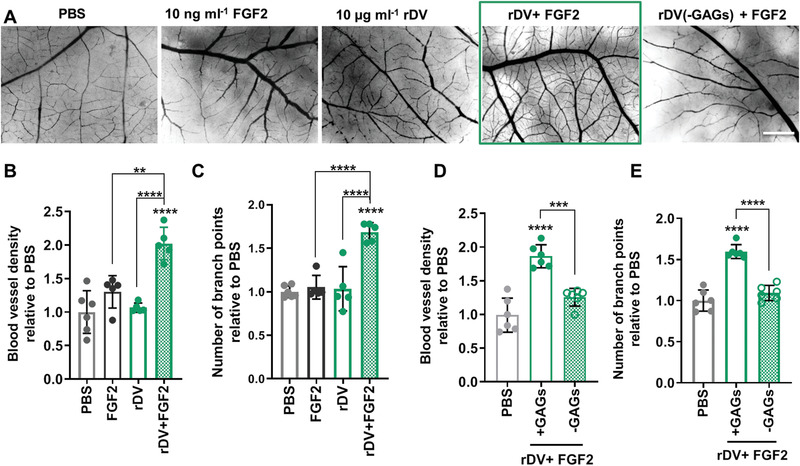
rDV promotes angiogenesis in vivo by potentiating growth factor signaling via its GAG chains. rDV was added to the CAM at embryonic day 8 (E8) and the effect on the vessel density and the number of branch points was studied at E12. A) Representative dissecting microscope images of the blood vessels in the CAM at E12 following exposure to PBS, FGF2 (10 ng mL^−1^), rDV (10 µg mL^−1^), or the two combined (rDV (10 µg mL^−1^) + FGF2 (10 ng mL^−1^)). Scale bar is 1 mm. B,C) Blood vessel density (B) and number of branch points (C) in CAM membranes exposed to PBS, FGF2 (10 ng mL^−1^), rDV (10 µg mL^−1^), or the two combined (FGF2 (10 ng mL^−1^)+ rDV (µg mL^−1^)). D,E) Blood vessel density (D) and number of branch points (E) in CAM membranes exposed to PBS or a combination of rDV and FGF2 (rDV (10 µg mL^−1^) + FGF2 (10 ng mL^−1^)) with GAG chains on rDV intact (+GAGs) or removed via enzymatic digestion (−GAGs). Data are expressed as fold change relative to PBS. Data are mean ± SD (*n* = 5–6). **p < 0.01 and ****p < 0.0001 relative to PBS unless indicated otherwise.

To test if rDV potentiates growth factor signaling, rDV and FGF2 were added to the CAM membrane at low concentrations (10 µg mL^−1^ and 10 ng mL^−1^, respectively) that did not show an effect on angiogenesis when added individually, but showed a significant (*p* < 0.0001) twofold increase in blood vessel density and a significant (*p* < 0.0001) 1.7‐fold increase in the number of vessel branch points compared to PBS (**Figure** **5**A–C). This indicates that rDV potentiates FGF2 signaling. To test if this effect was GAG‐chain mediated, GAG chains were enzymatically digested from the protein core prior to rDV addition to the CAM membrane. In the absence of GAG chains, rDV did not potentiate FGF2 signaling (*p* > 0.05 compared to PBS) (Figure 5D,E and Figure S1, Supporting Information).

### 2.3 rDV Promotes Vascularization of Implanted Biomaterials In Vivo

**Figure 6:**
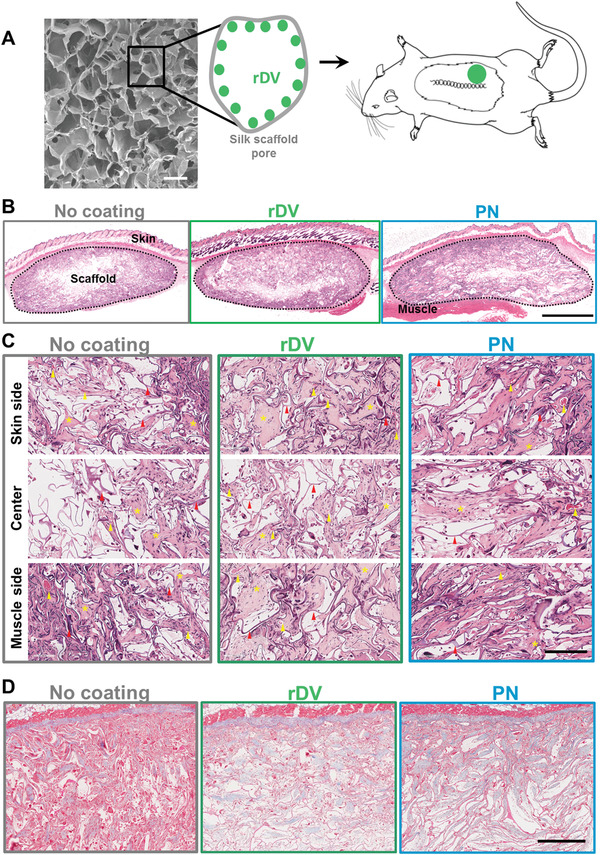
rDV supports tissue infiltration and integration of implanted silk fibroin biomaterials. 3D porous silk fibroin scaffolds were coated with rDV or full‐length endothelial cell perlecan (PN) and implanted subcutaneously in mice for 6 weeks. A) Representative SEM micrograph of the silk fibroin scaffold prior to implantation and schematic representation of the biomaterial coating and implantation. Scale bar is 200 µm. B) Representative gross morphological appearance of explanted silk fibroin scaffolds in the absence and presence of rDV or PN coating stained with H&E. The dashed line indicates the scaffold boundaries. Scale bar is 2 mm. C) The morphology of different anatomical regions within the implanted scaffold showing representative scaffold areas close to the mouse skin, in the center of the scaffold, and close to the mouse back muscles stained with H&E. Red arrows point to examples of silk scaffold lamellae, yellow arrows to blood vessels, and yellow asterisks to areas of ECM deposition. Scale bar is 200 µm. D) Collagen deposition in silk fibroin scaffolds in the absence of coating or when coated with rDV or PN stained with Masson's trichrome. Collagen is shown in blue, while silk lamellae and cells are pink. Scale bar is 500 µm.

To test the ability of rDV to promote angiogenesis and tissue vascularization in vivo, 3D porous silk fibroin scaffolds were coated with either rDV or perlecan via passive adsorption or uncoated, and implanted subcutaneously in mice for 6 weeks (**Figure** **6**A). Scaffolds were well‐tolerated and integrated with the surrounding tissue in all conditions as demonstrated by tissue ingrowth from the periphery toward the middle of the implanted scaffolds and very few foreign body giant cells observed throughout the implanted constructs (Figure 6B). A thin fibrous capsule (5–6 cell layers) was observed surrounding all constructs, but it did not prevent tissue infiltration, as cells infiltrated between the silk scaffold lamellae and tissue was deposited on and between the lamellae (Figure 6B). Analysis of different anatomical regions of the scaffold (close to the mouse skin, center of the scaffold, and close to the mouse skeletal muscle) further confirmed the presence of tissue between silk lamellae (Figure 6C). Qualitative analysis of the tissue from H&E (Figure 6C) and Masson's trichrome (Figure 6D) stains suggested that the tissue deposited in the presence of rDV and perlecan was less cellular compared to uncoated samples. All conditions supported deposition of collagen (Figure 6D) throughout the construct.

**Figure 7:**
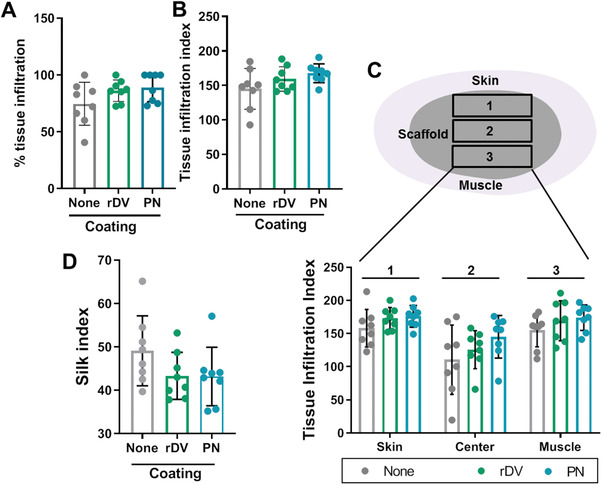
rDV does not affect tissue infiltration into implanted silk fibroin biomaterials. 3D porous silk fibroin scaffolds were coated with rDV or full‐length endothelial cell perlecan (PN) or uncoated (None), and implanted subcutaneously in mice for 6 weeks. A) Tissue infiltration into silk scaffold presented as percentage of overall scaffold area containing mouse tissue quantified from H&E stained images. B) Tissue infiltration index scored from H&E images across the entire scaffold. C) Tissue infiltration index scored from H&E images in different anatomical locations across the scaffold, including the skin side, scaffold center, and the muscle side. D) Silk index scored from H&E images across the entire scaffold showing the presence of silk scaffold lamellae. Data are mean ± SD (*n* = 8).

Quantification of the tissue infiltration as percent of overall scaffold area infiltrated by mouse tissue demonstrated 74.7 ± 18.9% tissue infiltration in the absence of coating and 86.2 ± 9.4% and 89.0 ± 12.4% in the presence of rDV and perlecan, respectively (**Figure** **7**A), showing no significant impact of coating on the overall tissue infiltration, and importantly no difference between rDV and perlecan. Tissue infiltration was further quantified and expressed as a ‘tissue infiltration index’ (Figure 7B) where 312 points across the construct were scored for the presence of tissue, giving a better indication of the overall tissue density within the scaffold, as opposed to gross percent tissue coverage. Tissue infiltration index was similar in the presence or absence of coating (*p* > 0.05), demonstrating that both rDV and perlecan support tissue infiltration. No significant differences were observed between different anatomical locations (Figure 7C), even though there was a trend toward a lower tissue infiltration index at the scaffold center compared to the skin or muscle side. Silk degradation was quantified by scoring the presence of silk lamellae at 312 locations across the construct to obtain a “silk index.” No significant difference in the silk index was observed between the different conditions (*p* > 0.05), even though there was a trend toward less silk scored in the presence of rDV or perlecan coating (Figure 7D).

**Figure 8:**
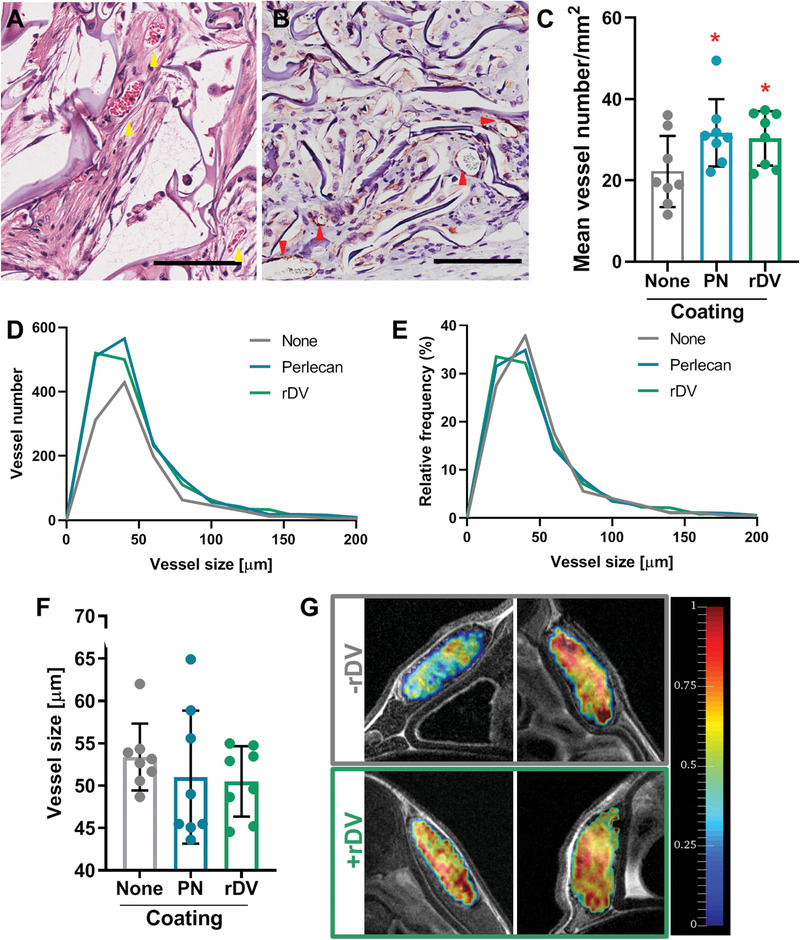
rDV promotes vascularization of implanted silk fibroin biomaterials. 3D porous silk fibroin scaffolds were coated with rDV, full‐length endothelial cell perlecan (PN) or uncoated (None), and implanted subcutaneously in mice for 6 weeks. A,B) Blood vessels in silk scaffolds coated with rDV. A) H&E and B) IHC *α*SMA. Red and yellow arrows point to representative blood vessels. Red blood cells appear red in H&E stained images. Brown color indicated *α*SMA staining, while blue/purple staining shows cell nuclei and silk scaffold lamellae. Scale bars are 100 µm. C) Blood vessel density in silk scaffolds quantified from histological images. Data are mean ± SD (*n* = 8). *p < 0.05 relative to ‘None’. D) Blood vessel size (vessel perimeter) distribution in silk scaffolds expressed as number of vessels per bin (20 µm bins). E) Blood vessel size (vessel perimeter) distribution in silk scaffolds expressed as relative frequency percent of each vessel size bin (20 µm bins). F) Mean blood vessel size (vessel perimeter). Data are mean ± SD (*n* = 8). G) Representative ΔR maps corresponding to relative blood volume inside silk scaffolds in the absence (−rDV) and presence (+rDV) coating observed via MRI imaging following injection of a contrast agent via a tail vein catheter. Top row are representative heat maps from two different animals implanted with uncoated scaffolds, while the bottom row are from two animals implanted with rDV‐coated scaffolds.

Blood vessel analysis was performed from histological images. Blood vessels were observed throughout the implanted silk scaffolds. Blood vessels in H&E stained sections showed an obvious lumen surrounded by a layer of cells and perfused with red blood cells (**Figure** **8**A). Cell periphery stained positive for *α*SMA demonstrating stable, mature vessels (Figure 8B). Quantitation of these histology images indicated a higher blood vessel density in the presence of rDV (*p* < 0.05) compared to no coating (Figure 8C). In the absence of rDV there were 22.2 ± 8.8 vessels mm^−2^, while in the presence of rDV there were 30.4 ± 6.7 vessels mm^−2^. Interestingly, blood vessel density was similar in scaffolds functionalized with rDV and those functionalized with full‐length human perlecan isolated from endothelial cells, (Figures 8C) demonstrating that this recombinant perlecan fragment supports a similar level of tissue vascularization to its full‐length counterpart. Blood vessel size (vessel perimeter) ranged from ≈10 to ≈600 µm with most vessels in the 10–100 µm range (Figure 8D,E). While analysis of overall vessel size frequency highlights the differences in vessel number between no coating and rDV or perlecan coating (Figure 8D), the relative frequency of different vessel sizes shows similar distribution patterns, with small differences in vessel sizes ≈20–60 µm (Figure 8E). There was no difference in the mean vessel size between different conditions (*p* > 0.05), with mean vessel size of 53.4 ± 3.9 µm in the absence of rDV and 50.5 ± 4.1 µm in the presence of rDV (Figure 8F). The vessels in the silk scaffolds were stable and perfused as demonstrated from histological sections. To further confirm the functionality and perfusion of the vasculature in the silk scaffolds, mice were injected with the Magnevist contrast agent via a tail vein catheter during MRI imaging. The contrast agent was observed in the silk scaffolds, indicating unobstructed delivery of blood via the vessels in the scaffold (Figure 8G).

## 3 Discussion

A potential means of overcoming limitations of angiogenic therapy, or stimulation of new blood vessel growth to treat ischemia using pro‐angiogenic factors, is through bio‐inspired approaches, in particular by mimicking the natural role of the ECM and GAGs in growth factor binding and signaling.^[^ ^6^ ^]^ One such example is demonstrated in the current study, where a recombinantly expressed C‐terminal fragment rDV of the proteoglycan perlecan is demonstrated to promote angiogenesis and vascularization of implanted biomaterials via GAG dependent growth factor signaling.

rDV was found to promote endothelial cell migration to the same extent as endothelial cell derived full‐length perlecan when immobilized to tissue culture plastic in an established in vitro endothelial cell wound healing model.^[^ ^22^ ^]^ Previous work has also demonstrated that rDV promotes the same level of endothelial cell adhesion and proliferation as full‐length perlecan.^[^ ^19^ ^]^ Further, in the current study, rDV was shown to promote endothelial cell sprouting, a critical aspect of the angiogenesis process,^[^ ^23^ ^]^ demonstrating that the effects of rDV are observed in both its immobilized and soluble forms. It was demonstrated that this process is GAG‐chain dependent, as the effects were lost in the absence of GAGs. We hypothesized that rDV promoted angiogenic sprouting via growth factors present in FBS which was provided in all cultures. This was confirmed when the addition of the anti‐FGF2 antibody resulted in a significant decrease in endothelial cell sprouting. It was interesting to note that addition of FGF2 to the 10% FBS control did not promote endothelial cell spouting in our studies, suggesting that rDV enhanced endogenous growth factor signaling at low growth factor concentrations, most likely by forming physiologically‐relevant tertiary complexes between FGF2, its cognate receptors, and HS chains on rDV, as has been demonstrated previously for full‐length perlecan.^[^ ^12a,b^ ^]^

While 2D and 3D in vitro endothelial cell assays are useful in probing aspects of the angiogenic process, they do not capture the complexity of angiogenesis observed in vivo. rDV was therefore tested in a CAM assay, a well‐established model of angiogenesis.^[^ ^24a,b^ ^]^ A range of substances have been found to promote angiogenesis in the CAM assay, including pro‐angiogenic growth factors FGF2 and VEGF165.^[^ ^24a^ ^]^ rDV was found to promote angiogenesis in a concentration dependent manner, with 10 µg mL^−1^ not increasing vessel density or number of branch points above the baseline, but doses of 20 and 50 µg mL^−1^ promoted a significant increase in both measures. rDV promoted an increase in angiogenesis similar to that of exogenously applied FGF2 at a dose of 50 ng mL^−1^. When rDV and FGF2 were combined at concentrations that were not individually able to promote an increase in angiogenesis (10 µg mL^−1^ rDV and 10 ng mL^−1^ FGF2), the combination resulted in a significant increase in both vessel density and number of vessel branch points, demonstrating that rDV potentiated FGF2 signaling at low concentrations. This effect was GAG chain dependent as removal of GAG chains from the rDV protein core via enzymatic digestion resulted in baseline angiogenesis levels. This is in line with the observations made with endorepellin (recombinantly expressed protein form of domain V region), which was found not to affect angiogenesis in the CAM assay.^[^ ^25^ ^]^ HS chains on full‐length endothelial cell derived perlecan have previously been demonstrated to form trimeric complexes with FGF2 and its cognate receptor FGFR1c, promoting FGF2 signaling.^[^ ^12b^ ^]^ FGF2 is found natively in the CAM and in the chorioallantoic fluid, with its concentration peaking between days 10 and 14.^[^ ^26^ ^]^ It is therefore likely that when rDV was added at higher concentrations (20 and 50 µg mL^−1^) it potentiated signaling of the endogenous FGF2, resulting in an increase in angiogenesis levels on par with that of exogenously added FGF2. These findings have implications for the potential therapeutic use of rDV, either as a means of potentiating endogenous growth factor signaling or lowering the effective therapeutic dose of exogenously delivered growth factors.

Vascularization of implanted bioengineered tissues or acellular biomaterials is critical to their survival and appropriate integration with the surrounding tissue.^[^ ^2^, ^27^ ^]^ While a range of strategies have been proposed toward improving vascular ingrowth into implanted constructs, no strategy to date has progressed to clinical application.^[^ ^2a^ ^]^ We investigated the potential of rDV to improve integration and vascularization of implanted silk fibroin scaffolds as biomaterial functionalization is one of the most accessible and clinically relevant strategies in terms of both the cost and regulation of medical devices. 3D porous silk scaffolds were functionalized with rDV via passive adsorption and implanted subcutaneously in mice. Silk scaffolds were well‐tolerated and integrated with the surrounding tissue, with cell ingrowth, ECM deposition, and minimal inflammatory response observed at 6 weeks post‐implantation. rDV did not affect the extent of tissue ingrowth, but the tissue in the presence of rDV appeared less cellular, suggesting rDV potentially promoted improved ECM deposition and tissue maturation. Despite a similar amount of tissue ingrowth, the number of blood vessels observed in rDV‐functionalized silk scaffolds was significantly higher than in the absence of rDV, suggesting rDV directly impacted on the extent of vascular ingrowth into implanted biomaterials. Similar results were observed with full length human perlecan indicating the ability of rDV to recapitulate its biological activity. Blood vessels in silk scaffolds were found to be mature and functional, with clear vessel lumens that stained for *α*SMA and were perfused with red blood cells. Additionally, when an MRI contrast agent Magnevist was injected into the tail veins of mice, the contrast agent was observed in the silk scaffolds, indicating unobstructed delivery of blood via the vessels in the scaffold.

It was interesting to note that rDV and full‐length endothelial cell perlecan elicited similar responses in these assays, suggesting that rDV can potentially be used in place of perlecan in therapeutic applications. This is important as rDV can be produced at higher yields and under defined culture conditions, and is easier to scale up relative to native perlecan production. rDV and full‐length perlecan in our studies elicited similar responses at the same working concentration (10–20 µg mL^−1^) despite their differences in molecular weight (460 kDa protein core for full‐length perlecan and 80 kDa for rDV). This is likely a result of differences in their structure, where perlecan has three HS chains per molecule, allowing GAG‐mediated growth factor signaling at lower molar concentration relative to rDV, which has one GAG chain (HS or CS) per molecule.^[^ ^19^ ^]^

Silk scaffolds in this study served as a model for tissue vascularization, but the effects are expected to be observed on other biomaterials. It is likely that just like in the endothelial cell and CAM assays, rDV promoted angiogenesis by potentiating signaling of the endogenous growth factors. Future work will also address the potential of rDV to potentiate exogenously delivered growth factor signaling in rodent models. Despite a mixed population of rDV (decorated with HS or CS GAG chains)^[^ ^19^ ^]^ being investigated in the current study, rDV performed in a similar manner to full‐length endothelial cell‐derived perlecan (decorated with three HS GAG chains on domain I)^[^ ^28^ ^]^ in terms of endothelial cell migration and implanted biomaterial vascularization. This may indicate that HS chains present in the subpopulation of rDV are sufficient to recapitulate the effects of full‐length perlecan or that CS plays a role in angiogenic growth factor binding and signaling.^[^ ^29^ ^]^ It would be of interest to isolate populations of rDV containing only HS or CS to test the effect of each population on growth factor signaling and angiogenesis. Metabolic engineering approaches toward expression of HS‐ or CS‐rich rDV populations are also of interest in particular as cell culture conditions are known to impact on the amount and the structure of GAG chains decorating proteoglycans.^[^ ^30a–c^ ^]^

In addition to the utility of its GAG chains in growth factor signaling, rDV and other proteoglycans carry additional functionalities in their protein core. ^[^ ^6^ ^]^ Perlecan protein core plays a major role in ECM and cell interactions,^[^ ^21^ ^]^ with the endothelial cell *α*2*β*1 integrin binding site is found in domain V. The protein core also provides a range of easily accessible chemistries that allow rDV to be immobilized on biomaterials such as silk. rDV immobilization can have a major effect on its presentation and function, as was demonstrated when rDV was immobilized on silk films.^[^ ^31^ ^]^ Control over the spatial orientation of growth factors or cell‐binding sequences has been shown to improve endothelial cell interactions and bioengineered tissue vascularization^[^ ^2a^ ^]^ and it would be of interest to develop spatial patterning of rDV on biomaterials for improved biological outcomes.

Finally, while silk biomaterials served as a vascularization model in this study, silk fibroin is also a highly promising biomaterial platform for a range of biomedical applications, including for musculoskeletal, skin, and vascular tissue engineering and regeneration.^[^ ^32^ ^]^ A number of silk materials, including surgical meshes, are FDA approved for medical applications.^[^ ^32^ ^]^ The aqueous silk solution can be engineered into films, fibers, porous scaffolds, micro‐ and nano‐particles, 3D printed structures, and hydrogels,^[^ ^33^ ^]^ and the 3D porous sponges used in this study have been utilized toward a number of soft tissue engineering applications.^[^ ^34^, ^35^ ^]^

## 4 Conclusions

Recombinantly expressed C‐terminal fragment of human perlecan rDV was shown to promote angiogenesis by potentiating growth factor signaling via its GAG chains. This finding has major implications for our understanding of perlecan biology, as well as potential therapeutic applications in the treatment of ischemic disease and vascularization of implanted biomaterials or bioengineered tissues. We envision delivery of rDV to either potentiate endogenous growth factor signaling or co‐delivery with exogenous growth factors to reduce their effective dose, providing efficacy, cost, and safety benefits for vascularization therapies.

## 5 Experimental Section

##### Materials

Bombyx mori silk cocoons were obtained from Tajima Shoji Co Ltd (Japan). C57BL/6 mice were sourced from Australian Bio Resources (Australia) and fertilized chicken eggs (Barter Black) were sourced from Barter and Sons Hatchery (Australia). Human umbilical cord endothelial cells (HUVEC) were sourced from Lonza (Australia). HEK‐293 cells stably transfected with rDV were established in‐house as previously described.^[^ ^18a^ ^]^ All other reagents were purchased from Sigma‐Aldrich unless stated otherwise.

##### Perlecan Expression and Isolation

rDV was expressed as previously described.^[^ ^18a^ ^]^ Briefly, domain V DNA (2446 bp, exons 79–97) was amplified from human coronary artery endothelial cell (HCAEC) mRNA, cloned into CEFLsec vector with a BM40 signal peptide, transfected into human embryonic kidney 293 (HEK‐293) cells using Lipofectamine 2000 (Thermo Fisher Scientific, USA) and stably transfected cells were selected using Geneticin. HEK‐293 cells were cultured in Dulbecco's Modified Eagle Medium with fetal bovine serum (FBS, 10% v/v) and penicillin‐streptomycin (1% v/v). Conditioned media were routinely collected for rDV purification via anion exchange chromatography on a diethylaminoethyl matrix as previously described.^[^ ^19^ ^]^ Proteoglycan yield was determined using the Bradford protein assay. Full‐length perlecan was immunopurified from the conditioned media of cultured HCAECs as previously described.^[^ ^36^ ^]^

##### Endothelial Cell Maintenance and Culture

HUVECs were cultured at 37 °C containing 5% CO_2_ in EBM‐2 Basal Medium supplemented with EGM Endothelial Cell Growth Medium SingleQuots Supplements (Lonza) as per manufacturer's instructions. Cells were passaged at 80% confluence and used at passage 3–8 in all experiments.

##### Endothelial Cell Migration Assay

Twelve‐well tissue culture plates were coated with rDV (10 µg mL^−1^), full‐length human perlecan (10 µg mL^−1^), or human fibronectin (10 µg mL^−1^) overnight at 4 °C and washed three times with phosphate buffered saline (PBS). Surfaces were blocked with freshly denatured (80 °C, 10 min) bovine serum albumin (BSA) for 30 min at 37 °C and washed twice in PBS prior to cell seeding. HUVECs were seeded at 5 × 10^4^ cells per well in cell culture medium until they reached 100% confluence. A 200 µL pipette tip was used to make a single vertical wound through the cell monolayer in each well. The medium was replaced, and plates were returned to the incubator. Three images of each well (top, middle, and bottom) were taken under a phase contrast microscope (4× objective) at different time points (0, 3, 8, and 24 h). HUVEC migration was plotted by calculating the percent of the original wound area covered by migrating cells at each time point using ImageJ (National Institutes of Health, USA).

##### Endothelial Cell Spheroid Sprouting Assay

To generate endothelial cell spheroids, HUVECs (passage 3–5) were suspended in cell culture medium with methyl cellulose (0.4% w/v, made using 2% w/v Methocel A4M stock dissolved in EBM‐2 Basal Medium, filter sterilized twice), seeded in non‐adherent round bottom 96‐well plates (Greiner CELLSTAR) at 750 cells per well, and cultured at 37 °C, 5% CO_2_ overnight. Spheroids were harvested and suspended in EBM‐2 Basal Medium (100 spheroids per milliliter) supplemented with FBS (10% v/v). To embed spheroids in fibrin hydrogels, thrombin (4 U mL^−1^), fibrinogen (4 mg mL^−1^) (Merck, USA) and media containing cell spheroids were combined in a 1:5:5 volume ratio. The solution (250 µL) was pipetted in 48‐well plates and allowed to polymerize for 15 min at room temperature, followed by 15 min at 37 °C. EBM‐2 Basal media (500 µL) containing FBS (10% v/v) and test substances of interest (VEGF165 (5 ng mL^−1^) or rDV (20 or 50 µg mL^−1^)) was added on top of the gels. Some experiments involved GAG chain removal from rDV. GAG chains were removed from the protein core prior to incubation with endothelial cell spheroids by incubation with chondroitinase ABC (0.05 U mL^−1^) and heparinase III (0.01 U mL^−1^) for 16 h at 37 °C to remove CS and HS chains, respectively. To test endogenous signaling by FGF2 in FBS, some experiments involved incubating bovine anti‐FGF2 antibody (R&D systems). Anti‐FGF2 antibody (2 mg mL^−1^) was incubated with FBS overnight prior to mixing with EBM‐2 Basal media to a final FBS concentration of 10% v/v, and addition on top of the gels. Gels were imaged using a phase contrast microscope (Olympus CKX41) at 10× magnification, and again after incubation at 37 °C and 5% CO_2_ for 24 h. Sprouting from the spheroid bodies was quantified by measuring the area of the entire spheroid including sprouts and subtracting the original spheroid area prior to sprouting using ImageJ.

##### Chicken Chorioallantoic Membrane Angiogenesis Assay

CAM assay experimental protocols were approved by the UNSW Animal Care and Ethics Committee (ACEC 17/36A). All surgical procedures were performed under aseptic conditions. Fertilized eggs were cleaned with 80% ethanol and incubated in a specialized chicken incubator (Multiquip, Australia) at 37.5 °C with 45–50% humidity. The eggs were rotated every 5 h for the first 3 days. The fertilization and viability of the chicken embryos were checked by candling at embryonic day 4 (E4). At E4, 3–4 mL of albumen was aspirated from the egg using a 19G needle to detach the CAM from the egg shell. A 1 cm^2^ window was opened in the side of the egg shell using scissors and the window was sealed with Aquafilm transparent dressing (Livingstone Laboratory Supplies, Australia) to prevent dehydration and possible infections before returning to the incubator. At E8, the window in the shell was reopened and a single 9 mm inner diameter silicone ring was placed on top of the CAM in each egg. 100 µL of each sample (*n* = 6 per condition) was pipetted into the silicone ring and the window was resealed with the Aquafilm transparent dressing prior to eggs being returned to the incubator. Samples included PBS (negative control), FGF2 (50 ng mL^−1^, positive control), and rDV (10‐50 µg mL^−1^). Some experiments utilized FGF2 at 10 ng mL^−1^ and rDV without GAG chains. GAG chains were removed from the protein core prior to incubation on the CAM by incubation with chondroitinase ABC (0.05 U mL^−1^) and heparinase III (0.01 U mL^−1^) for 16 h at 37 °C to remove CS and HS chains, respectively. At E12, the CAM membrane was excised and the area inside the silicone ring was imaged using a dissecting microscope. Blood vessel density was quantified from each captured image using image processing implemented in the Matlab language (R2017b, Mathworks, USA). The raw image was first decomposed into the red, green, and blue channels. The red channel was then zeroed and contrast stretching was performed on the green and blue channels individually such that the bottom 1% and the top 1% of all pixel values were mapped to between 0 and 1, respectively. The resulting image was converted to grayscale by eliminating the hue and saturation information while retaining the luminance. Finally, a median filter with a 3 × 3 pixel kernel was applied to the resulting grayscale image, which was again followed by contrast stretching, resulting in the pre‐processed image. A minimum (S_min_) and maximum (S_max_) feature size in pixels were input by the user. For each feature size between S_min_ and S_max_, in steps of two pixels, morphological bottom‐hat filtering was applied to the pre‐processed image with a disk‐shaped structuring element with radius equal to the feature size. A threshold, determined using the Otsu method,^[^ ^37^ ^]^ was then applied to the resulting image to produce a logical image where the pixel value at each location is true if that pixel belonged to a feature that is the same size as the current feature size, or false otherwise. Finally, a logical OR operation was applied to the logical images for each feature size to generate the final logical image, in which a pixel with a value of true represents the positive identification of a blood vessel. The number of true pixels in the final logical image represents the amount of vascularization and is presented as fold‐change relative to the negative control (PBS). The number of blood vessel branch points was manually counted using ImageJ.

##### Silk Fibroin Isolation and Scaffold Fabrication

Silk fibroin solution was prepared as previously described.^[^ ^33^ ^]^ Briefly, Bombyx mori silk cocoons were cut into 2 cm^2^ pieces and boiled in sodium carbonate solution (0.02 m, 2.5 g L^−1^ sodium carbonate) for 30 min to remove sericin. Silk fibroin fibers were washed in water and dissolved in lithium bromide (9.3 m, 4 mL g^−1^ silk fibers) for 4 h at 60 °C. The solution was dialyzed against water using SnakeSkin dialysis tubing (3500 MWCO, Thermo Fisher Scientific, USA) for 3 days. The concentration of the silk solution (6–8% w/v) was determined by drying a known volume and weighing the remaining film. Silk fibroin solution was stored at 4 °C. Porous silk scaffolds were fabricated by freezing the silk solution (2% w/v) in 24‐well tissue culture plates overnight at −20 °C, followed by lyophilization and autoclaving at 121 °C to induce *β*‐sheet formation, as previously described.^[^ ^34^ ^]^ Scaffolds were hydrated in PBS and cut to 8 mm (diameter) × 2 mm (height) discs. Scaffold pore morphology was visualized using scanning electron microscopy (SEM). Briefly, scaffolds were sequentially dehydrated in ethanol using a microwave processor (PELCO BioWavePro+, Ted Pella, USA) and dried using a critical point dryer (Autosamdri‐815, Tousimis, USA). Samples were coated with platinum (K575X, Emitech, France) and imaged using a Hitachi SEM3400I. Scaffolds were coated with rDV or full‐length endothelial cell perlecan by incubating scaffolds with rDV/perlecan (20 µg mL^−1^) overnight at 4 °C as previously described.^[^ ^20^ ^]^

##### Subcutaneous Implant Assay

All procedures were conducted in accordance with animal ethics protocol (ACEC 17/2B) approved by the UNSW animal ethics committee on 8‐week‐old female C57BL/6 mice. The back of each mouse was shaved and the surgical site disinfected 3X with betadine swabs followed by isopropanol wipes under general anesthesia of isoflurane. Buprenorphine (0.1 mg kg^−1^) analgesia was administered pre‐operatively. Silk scaffolds were implanted in subcutaneous pockets on the back of each mouse (*n* = 8 per condition) for 6 weeks as previously established.^[^ ^34^, ^38^ ^]^ The incisions were closed with surgical clips that were removed at day 10 post‐surgery. At week 6 post‐surgery the mice were processed for either histological or magnetic resonance imaging (MRI) analyses.

##### Histological and Immunohistochemical Analysis of Explanted Silk Scaffolds

Silk scaffolds and surrounding tissue were excised from the animals following euthanasia and fixed in neutral buffered formalin (10% v/v). Scaffolds were cut in half along the vertical axis and embedded such that the center of the scaffold was sectioned and analyzed. Samples were embedded in paraffin and sectioned to 4 µm thickness using a microtome. Samples were deparaffinized and rehydrated using a series of xylene and graded ethanol incubations. Histological analyses were carried out using hematoxylin and eosin, Masson's trichrome, and immunohistological staining of *α*‐smooth muscle actin (*α*SMA) as previously described.^[^ ^39^ ^]^ Briefly, slides were stained with Harris haematoxylin (Thermo Fisher Scientific, USA) for 10 min, followed by eosin (Thermo Fisher Scientific, USA) for 5 min, before dehydration and mounting. Masson's trichrome staining was performed following manufacturer's standard procedure. Briefly, samples were mordanted in preheated Bouin's solution at 56 °C for 15 min and washed with tap water. The samples were then stained with Hematoxylin solution Gill No. 3 (Vector Laboratories, USA) for 5 min, Biebrich Scarlet‐Acid Fucshin for 5 min, Phosphotungstic/Phosphomolybdic Acid solution for 5 min, and last Aniline Blue solution for 5 min with 5 min tap water washes in between each stain. The samples were placed in acetic acid (1% v/v) for 2 min before dehydration and mounting.

For immunohistochemical analysis, antigen epitope retrieval was performed by immersing the slides in sodium citrate (0.01 m, pH 6), followed by heat treatment in a decloaking chamber (Applied Medical, USA) at 120 °C for 4 min. Slides were rinsed with Tris‐buffered saline (TBS, Tris (0.05 m) NaCl (0.15 m), pH 7.6) followed by blocking with goat serum (10% v/v) in TBS for 1 h at room temperature. The slides were incubated with a primary anti‐*α*SMA antibody (Abcam, Product ID: ab5694, 1:1000) with goat serum (1% v/v) in TBS at 4 °C for 16 h. Slides were washed twice with TBST (TBS with Tween 20 (0.05% w/v)) before incubating with a biotinylated anti‐rabbit secondary antibody (GE Healthcare, 1:500) for 1 h at room temperature. The slides were washed twice with TBST then incubated for 30 min with streptavidin‐HRP (1:250), rinsed four times with TBST before color development with NovaRED chromogen stain (Vector Laboratories, USA). Slides were then counterstained with Hematoxylin solution Gill No. 3 for 10 s and rinsed with deionized water. All samples were imaged using Aperio ScanScope XT scanner.

Scaffold vascularization was quantified from 16 H&E stained images (858 µm × 465 µm) captured from a single section at the center of each sample. The images represented different anatomical locations across the scaffold, including six from the side in contact with the mouse skin (skin side), six from the side in contact with the mouse muscle (muscle) side, and four from the middle of the scaffold section.

Blood vessel number and size were measured by manually tracing out the perimeter of every blood vessel (identified through a clear lumen and perfusion with red blood cells) using ImageJ present in each image (16 images per sample, *n* = 8 samples per condition). Tissue infiltration into implanted silk scaffolds was analyzed by either tracing the overall area of tissue infiltration in each whole scaffold section (*n* = 8 per condition) and determining the percentage of the total scaffold area that had tissue ingrowth, or by determining the “tissue infiltration index.” Tissue infiltration index was calculated by overlaying each image (16 images described above per sample) with a 24 × 13 grid and at each grid intercept scoring for the presence of infiltrated tissue. Silk degradation was assessed from the same histological images by scoring for the presence of silk scaffold lamellae (“silk index”). The scores for each image were expressed as percentage of overall number of points scored (312).

##### Analysis of Scaffold Vascularization Using Magnetic Resonance Imaging

Prior to imaging animals were catheterized with a 30G needle tip attached to polyethylene tubing through the tail vein under isoflurane anesthesia and appropriate liquid flow was verified by injection of 50 µL of saline. Animals were imaged using a 9.4‐T Bruker BioSpec 94/20 Avance III micro‐imaging system (Bruker, Germany) equipped with BGA‐12S HP gradients with maximum strength 660 mT m^−1^ and slew rate 4570 Tm s^−1^. A volume quadrature transmit coil with ID 86 mm was used for signal excitation and signal was received by a 20 mm ID single loop surface coil, attached directly to the mouse back with the scaffold of interest. The imaging protocol comprised high resolution T2w imaging for anatomical reference and pre‐ and post‐contrast T1 mapping to quantify R1 relaxivity changes as a surrogate marker for overall blood volume inside the scaffold. Structural information was acquired using an optimized 2D T2w fast spin echo (TurboRARE, TSE) sequence in axial orientation. The imaging slab contained 15 2D slices of 300 µm thickness (100 µm gap) with a total coverage of 6 mm, covering the entire scaffold. Major Imaging parameters were: TR = 2200 ms, TE = 33 ms, ETL = 8, FOV 192 × 192 mm, matrix size 256 × 256, in‐plane resolution 75 × 75 µm, total acquisition time 11 min. T1 relaxivity was quantified pre‐ and post‐contrast injection using a multi TR TSE saturation recovery sequence with six recovery times, optimized to cover the expected recovery curve in the scaffold. Major sequence parameters for the T1 mapping sequence were: TE = 10 ms, TR = [200, 400, 800, 1500, 3000,7500 ms], ETL = 2, FOV 192 × 192 mm, matrix size 64 × 64, in‐plane resolution 300 × 300 µm, total acquisition time 10 min. For the post‐contrast relaxivity measurement a 100 µL bolus of GdDTPA (0.2 mol kg^−1^, Magnevist, Bayer, Germany) was injected via the tail vein catheter directly after the first pre‐contrast T1 scan. Post‐contrast T1 was quantified at a single time point by starting a second T1 acquisition with identical calibration exactly 10 min after bolus injection.

Relative change in blood volume inside the silk scaffolds was quantified by calculating the difference in tissue R1 relaxation rates between pre‐ and post‐contrast map that was used as a well‐established surrogate marker. Pre‐ and post‐contrast relaxation rates are connected by a simple linear relationship R1_post_ = R1_pre_+r1[CA], where r1 is the inherent relaxation rate and [CA] the concentration of the specific contrast agent used.^[^ ^40a,b^ ^]^ As a blood volume marker local Δ*R* = r1[CA] = R1_post_‐R1_pre_ was quantified as it directly reflects the amount of contrast agent tracer delivered into the scaffold via the blood stream. Data evaluation was performed using in‐house developed Python software, based on ITK and numpy image processing packages. T1 relaxivity maps were calculated for both pre‐ and post‐contrast scans by pixelwise fitting a saturation recovery curve to the signal‐time series. Binary scaffold masks were generated by delineating the entire scaffold using the 3D Slicer package and exported in nifti. The masks were co‐registered to calculate an affine transformation between pre‐ and post‐scans, which was then used to align the pre‐ and post‐T1 relaxivity maps for maximum overlap. R1 maps were then calculated by inversion of the co‐registered maps and subtracted in the overlap region to obtain a ΔR map for each scan timepoint. Maps were overlayed on the structural scans for anatomical references.

##### Statistical Analyses

Statistically significant differences were determined by *t*‐test, or one‐ or two‐way analysis of variance (ANOVA) and the Tukey post‐test using GraphPad Prism. Data are expressed as mean ± standard deviation (SD) unless stated otherwise. Statistical significance was accepted at *p* < 0.05 and indicated in the figures as *p < 0.05, **p < 0.01, ***p < 0.001, and ****p < 0.0001.

## Conflict of Interest

The authors declare no conflict of interest.

## Supporting information

Supporting Information
