# Supplementary material for: A Biomimetic Approach toward Enhancing Angiogenesis: Recombinantly Expressed Domain V of Human Perlecan Is a Bioactive Molecule That Promotes Angiogenesis and Vascularization of Implanted Biomaterials
Source: Adv Sci (Weinh). 2020 Jun 14;7(17):2000900. doi: 10.1002/advs.202000900 (PMC7507460; doi:10.1002/advs.202000900)
Supplement: Supplementary file 1 — Supporting Information [file ADVS-7-2000900-s001.pdf]

Supplementary Data

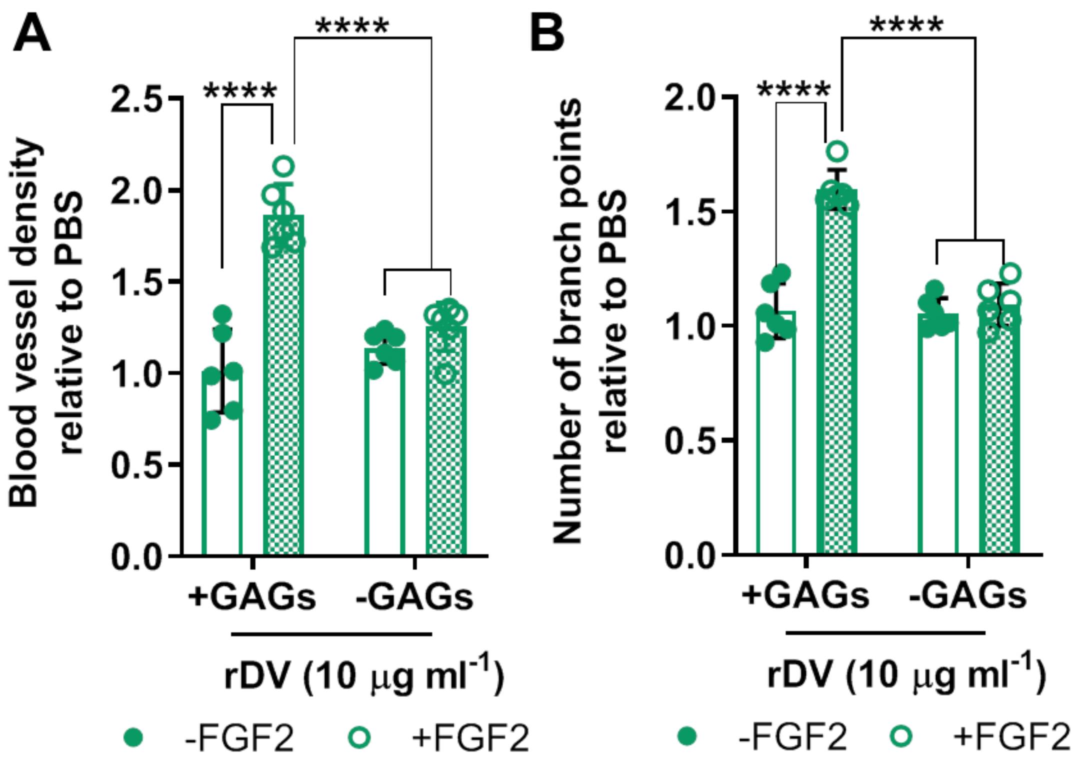

Figure S1. Perlecan domain V (rDV) promotes angiogenesis *in vivo* by potentiating growth factor signalling via its GAG chains. rDV was added to the chicken chorioallantoic membrane (CAM) at embryonic day 8 (E8) and the effect on the vessel density and the number of branch points was studied at E12. **(A)** Blood vessel density and **(B)** and number of branch points in CAM membranes exposed to 10 µg/ml rDV with (+GAGs) or without (-GAGs) chains in the absence (-FGF2) or presence (+FGF2) of 10 ng ml^{-1} FGF2. This figure complements Figure 5, showing all the relevant controls and demonstrating that at 10 µg ml^{-1} rDV does not promote angiogenesis in the absence of FGF2, regardless of the presence of GAG chains. Data are expressed as fold change relative to PBS. Data are mean±SD (n=5-6). ** (p<0.01) and **** (p<0.0001) as indicated in the figure.
